# Calcium Salts in Ulcers of the Legs and in Chilblains

**Published:** 1907-08-17

**Authors:** 


### Points in Treatment

### CALCIUM SALTS IN ULCERS OF THE LEGS AND IN CHILBLAINS.

Much has been written of recent years regarding the beneficial effects of calcium salts in many different conditions, and we have already expressed the view that preparations of calcium will in all probability be used in unsuitable cases, and so may very likely fall into disrepute.

The best safeguard against such disrepute is to collect together those conditions in which the administration of calcium salts has been shown to be of undoubted benefit. The fact that any particular case does well upon a particular drug is always of interest, but to test the efficacy of a medicine it is essential to try it in a very large number of cases all exhibiting similar pathological lesions. If the lesions, upon the whole, heal sooner when the medicine is given than when it is not, the conclusion must be that the drug is a useful one in promoting the cure of that particular affection. There will still be individual variations, of course; and although the drug may do good to A, B, C, and D, it may have no such effect on E.

### Trophic Disturbances.

There is strong evidence that two well-defined pathological lesions whose cure is greatly assisted by giving calcium salts are:

1. Chronic "simple" ulcers of the legs.
2. Chilblains.

Both of these come under that vague pathological heading to which the term, "trophic disturbances," is applied; the calcium salts can hardly be expected to cure the varicose veins, for example, to which ulceration of a leg may be indirectly due; but they most certainly improve the general condition of the soft parts, rendering the dys-trophy less marked, with the result that the actual lesions of the skin exhibit increased recuperative power and a spontaneous tendency to heal. In the case of chilblains it is very possible that there is not only an increase in the power of the tissues to maintain, or recover, their normal nutrition, but also a direct effect upon the vessel walls or upon the nerves controlling them, so that the local circulation is less readily upset by changes in the external temperature.

The results obtained are very clear, however the calcium salts bring them about. The two compounds that are of most service are the chloride and the iodide, either of which may be given in doses of from two to ten grains. The iodide is the more expensive, but it is said to be rather the more efficacious; probably the iodine has an action in addition to the calcium, but it is to the latter that the chief action is due, for not only will the chloride do almost as well as the iodide, but also the giving of iodide of potassium has nothing like the effect of giving iodide of calcium, except in tertiary syphilitic cases. In some cases of chronic simple ulceration of the leg in which iodide of potassium had been given over a long period without avail, the administration of calcium iodide has been immediately followed by slow but steady repair of the ulcer and comparatively rapid recovery.

Poor-law infirmaries and similar institutions are admirable places for testing the drug, and there are already several reports as to the benefits obtained. To an inmate of an infirmary the possession of a severely ulcerated leg has hitherto been practically equivalent to a home therein for life. This is ceasing to be the case now that calcium iodide in two-grain doses is being used; ulcers that have stubbornly resisted other treatment for years have been found to present healthy granulating surfaces in a few weeks after this drug is begun; the surrounding induration becomes greatly diminished, and ultimately the ulcer, which would never heal before, becomes entirely covered with healthy skin and is cured. It is even reported that some chronic ulcer patients have been so alarmed at the way their ulcers were healing up that they have taken active steps to promote extension of the ulcer and to counteract the good effects of the calcium iodide.

The other usual forms of treatment by fomentations in the septic cases, rest in bed, and so on, will naturally not be dispensed with as soon as iodide of calcium is given; the latter is a remedy to be used in addition to, and not in place of, the others, and as another string to the medical practitioner's bow it is a confirmed success.

### Chilblains.

Chilblains are not likley to come under treatment at this time of year, but when the winter begins it will be well worth while to bear in mind the benefits to be obtained from the internal administration of calcium chloride in patients who are tormented by them. Many people regard their chilblains as inevitable, and they just put up with them, struggling along without seeing any doctor, notwithstanding the fearful itching, the pain, and the actual sores which arise if the blains burst. They think that the doctor does them no more good than they can do for themselves, and in the past this has been so except when actually septic blains have required treatment. A medicine which will cure chilblains, or even relieve them, must be a boon to patient and doctor alike; such a medicine is calcium chloride. It may be given as a prophylactic, on the principle that prevention is better than cure. A better test of its efficacy is its administration when the chilblains are present and severe. In many cases it acts almost like a charm. We have seen patients whose fingers and toes were all bloated and disfigured by the blains, some of which were burst, recover completely in a week under conditions when, from their previous experience, they would have gone on suffering for months if the medicine had not been taken. There seems to be no necessity to treat the condition locally at all; unless, that is to say, some infection with pyogenic organisms requires boric acid fomentations or the like. The chilblains themselves subside spontaneously when calcium chloride is given by the mouth, and before the blains have themselves gone the patient has received relief to the distressing symptoms of itching and pain.

